# Development of an Interactive AI System for the Optimal Timing Prediction of Successful Weaning from Mechanical Ventilation for Patients in Respiratory Care Centers

**DOI:** 10.3390/diagnostics12040975

**Published:** 2022-04-13

**Authors:** Kuang-Ming Liao, Shian-Chin Ko, Chung-Feng Liu, Kuo-Chen Cheng, Chin-Ming Chen, Mei-I Sung, Shu-Chen Hsing, Chia-Jung Chen

**Affiliations:** 1 Department of Pulmonary Medicine, Chi Mei Medical Center, Chiali, Tainan 72263, Taiwan; 980897@mail.chimei.org.tw; 2 Department of Respiratory Therapy, Chi Mei Medical Center, Tainan 710402, Taiwan; 737005@mail.chimei.org.tw (S.-C.K.); mayyi323@gmail.com (M.-I.S.); rtlisa1965@gmail.com (S.-C.H.); 3 Department of Medical Research, Chi Mei Medical Center, Tainan 710402, Taiwan; 4 Department of Internal Medicine, Chi Mei Medical Center, Tainan 710402, Taiwan; 5 Department of Intensive Care Medicine, Chi Mei Medical Center, Tainan 710402, Taiwan; chencm3383@gmail.com; 6 Department of Information Systems, Chi Mei Medical Center, Tainan 710402, Taiwan; carolchen@mail.chimei.org.tw

**Keywords:** artificial intelligence, machine learning, weaning timing, successful weaning, prediction, mechanical ventilation, respiratory care center, dashboard, impact analysis

## Abstract

Successful weaning from prolonged mechanical ventilation (MV) is an important issue in respiratory care centers (RCCs). Delayed or premature extubation increases both the risk of adverse outcomes and healthcare costs. However, the accurate evaluation of the timing of successful weaning from MV is very challenging in RCCs. This study aims to utilize artificial intelligence algorithms to build predictive models for the successful timing of the weaning of patients from MV in RCCs and to implement a dashboard with the best model in RCC settings. A total of 670 intubated patients in the RCC in Chi Mei Medical Center were included in the study. Twenty-six feature variables were selected to build the predictive models with artificial intelligence (AI)/machine-learning (ML) algorithms. An interactive dashboard with the best model was developed and deployed. A preliminary impact analysis was then conducted. Our results showed that all seven predictive models had a high area under the receiver operating characteristic curve (AUC), which ranged from 0.792 to 0.868. The preliminary impact analysis revealed that the mean number of ventilator days required for the successful weaning of the patients was reduced by 0.5 after AI intervention. The development of an AI prediction dashboard is a promising method to assist in the prediction of the optimal timing of weaning from MV in RCC settings. However, a systematic prospective study of AI intervention is still needed.

## 1. Introduction

Respiratory failure is a critical condition that occurs when the lungs cannot obtain enough oxygen or when carbon dioxide is retained in the body, resulting in tissue hypoxemia and hypercapnia. This can be caused by a direct injury to the pulmonary tissue or by other systemic diseases that affect breathing [1]. According to Carson et al. [2], the incidence, mortality, and medical expenditure related to respiratory failure increase with age.

Patients with respiratory failure need to receive invasive mechanical ventilator therapy. In the US, it was estimated that there were 2.7 episodes of mechanical ventilation per 1000 population, with a national cost of approximately $27 billion, which constitutes 12% of all hospital costs [3].

Patients with acute respiratory failure who receive invasive mechanical ventilation during the critical stage can still be successfully weaned after recovery from the precipitating illness. Some patients need a longer period of weaning in order to recover. The National Association for Medical Direction of Respiratory Care defined prolonged mechanical ventilation (MV) as the administration of mechanical ventilation for more than 21 days and more than 6 h per day [4]. In Taiwan, the National Health Insurance established a transfer system in which ICU patients under prolonged MV (>21 days) are transferred and cared for in respiratory care centers (RCCs) until they are weaned from the ventilator. In RCC, the early detection of some level of independent respiration in patients is important to allow them to progressively wean from the ventilator until their respiratory systems function independently. Unfortunately, there is a lack of reliable MV-weaning decision-making tools that consider the complex states of patients to provide objective, scientific, and successful weaning probabilities for medical personnel to use. As a result, medical personnel may underestimate patients’ ability to recover; thus, attempts at weaning may be delayed, which not only increases medical costs but also causes patients discomfort.

Artificial intelligence (AI) or machine-learning (ML) approaches are complicated modeling techniques that can model extremely complex functions, often with higher predictive model quality than traditional statistics [5]. They can be applied in many situations in which a relationship between independent variables (inputs) and dependent variables (outputs) exists. AI or ML methods provide great opportunities to improve patient outcomes. ML methods have been applied to help clinical decision making by using a large amount of digital information generated in medical settings. However, using AI or ML technologies in the prediction of the timing of the weaning of patients from MV is quite rarely studied in the RCC setting.

Our study aimed to develop an interactive AI system for the optimal timing prediction of successful weaning among patients who received invasive MV while in a RCC. A preliminary AI intervention analysis was conducted as well. The main contributions of this research are: (1) the use of an AI approach for modeling MV-weaning prediction in RCCs with clinically available resources; (2) the development of an AI model into an innovative prediction system to assist RCC clinical decision making; and (3) the implementation of a preliminary AI impact analysis to demonstrate the clinical benefits of AI.

The rest of this paper is structured as follows: Section 2 gives an overview of related literature; Section 3 presents how the data were collected and how the study was conducted; Section 4 presents the results obtained in this study; Section 5 discusses the outcomes and findings of the study; and finally, Section 6 provides conclusions and directions for further research.

## 2. Related Work

A previous study used respiratory pattern variability in patients during the weaning process to determine the differences between patients who could and could not tolerate a spontaneous breathing trial; however, only 64 patients were enrolled in AI modeling [6]. Ossai and Wickramasinghe [7] reviewed 26 papers and found that there is a progressive interest in methods that could reinforce the management of patients under prolonged MV due to the increased medical costs and associated adverse outcomes. They pointed out that the small data size and poor study design hampered the development of a unified approach to managing MV in the ICU. They also concluded that the ensemble model predicted ventilator weaning better than other algorithms.

Kwong et al. [8] conducted a systematic literature review and meta-analysis of studies on weaning from MV that used AI and ML in ICUs. They identified nine studies that used AI/ML to predict ventilator weaning. Their management included the prediction of arterial blood gases, successful spontaneous breathing trials, successful extubation, ventilator setting, and oxygenation status. The study found that an insufficient number of studies have begun to evaluate the efficacy and effectiveness of AI and ML for ventilator weaning in ICUs. The authors suggested that AI and ML have an important role in predicting spontaneous breathing trial failure, extubation failure, arterial blood gases, and the adjustment of MV settings to determine levels of oxygenation. The available studies for the application of AI and ML in this area may still be limited.

One of our previous AI/ML works developed an AI model for predicting risk before lung resection surgery to provide information for anesthesiologists on whether a patient can be extubated immediately after surgery, because it is difficult for doctors to comprehensively evaluate risk factors to assess patients in time-limited pre-anesthetic clinics [9]. In this previous study, we developed a predictive system to demonstrate the feasibility of an AI model; however, the benefit of the AI intervention was not explored. Based on the review of related works, an AI model was used to develop a system based on electronic medical data that can predict whether patients could be weaned immediately after lung resection surgery [9].

## 3. Materials and Methods

### 3.1. Ethical Consideration

The present study was approved by the Institutional Review Board of the Chi Mei Medical Center (IRB Serial No.: 10912-016). All methods were carried out in accordance with relevant guidelines and regulations. Informed consent from patients was waived due to the retrospective nature of the study.

### 3.2. Study Design

**Figure 1:**
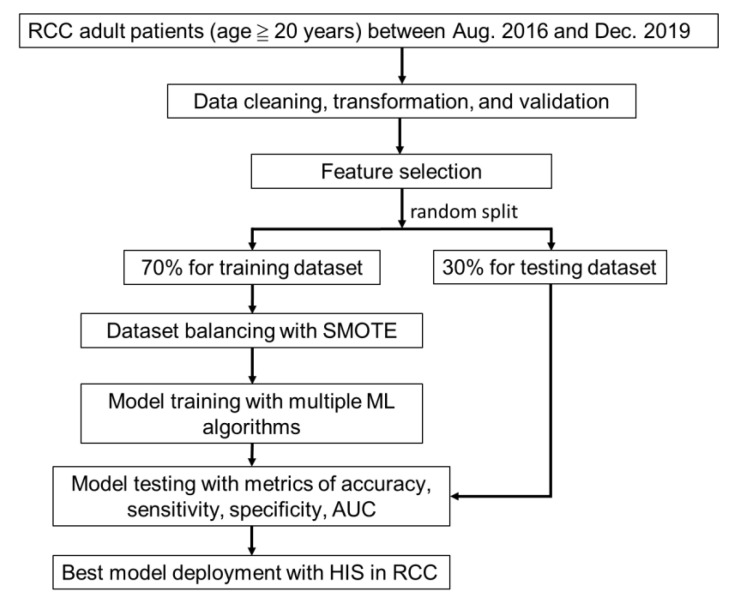
Research Flow. RCC: respiratory care center; SMOTE: synthetic minority oversampling technique; AUC: area under the receiver operating characteristic curve; HIS: hospital information system.

We organized a multi-disciplinary team, including physicians, respiratory therapists, data scientists, and information engineers, for this study. Adult patients (age ≥ 20 years) who had a tracheotomy or endotracheal intubation, and were admitted to the RCC wards of Chi Mei Medical Center from August 2016 to December 2019 were enrolled in the present study. Figure 1 shows the research flow.

### 3.3. Feature and Outcome Variables

The 26 feature variables included for modeling were age, gender, APACHE II (Acute Physiology and Chronic Health Evaluation II) score in RCC, GCS_E (Glasgow Coma Scale_eyes opening) in RCC, GCS_M (Glasgow Coma Scale_motor response) in RCC, heart rate, SBP (systolic blood pressure), DBP (diastolic blood pressure), suction times, duration of MV (periods in support mode), FiO2, PEEP (positive end-expiratory pressure), RR Actual (respiratory rate actual), MV Actual (minute ventilation actual), mPaw (mean airway pressure), SpO2, PSL (pressure support level), PSL volume, T-piece trial count, and presence of comorbidities such as diabetes, chronic obstructive pulmonary disease (COPD), myocardial infarction (MI), stroke, end-stage renal disease (ESRD), pneumonia, and sepsis. These features were chosen because of their wide availability in the RCC setting. All feature variables were extracted from the hospital information system (HIS) database as well as real-time recordings of ventilator outputs. Referring to a Taiwanese population-based study [10], we defined the outcome variable of successful weaning as weaning from MV for five consecutive days. This is also accepted as a basis for the provision of government-related health subsidies in Taiwan. Meanwhile, failed weaning included transfer back to ICU/ward with MV, transfer to respiratory care ward (RCW, long-term care settings to which prolonged mechanical ventilator (PMV)-dependent RCC patients can be transferred), discharge from hospital in critical condition (critical AAD), and death. The outcome variable was binary-coded with 1 as successful weaning and with 0 as failed weaning.

### 3.4. Model Building and Measurement

Before modeling, the data were randomly split into the training dataset (70%) and the test dataset (30%). To balance the dataset with an imbalanced outcome class, the synthetic minority over-sampling technique (SMOTE) [11] was applied. SMOTE is an oversampling technique in which the minority class is oversampled by creating “synthetic” samples along the line segments joining any or all of the minority class’ nearest neighbors. This technique was adopted only for the training dataset.

We used seven supervised ML algorithms, which included logistic regression (LR), random forest (RF), support vector machine (SVM), k-nearest neighbors (KNN), extreme gradient boosting (XGBoost), light gradient boosting machine (LightGBM), and multilayer perceptron (MLP), to build the models and measure the models’ quality in terms of accuracy, sensitivity, specificity, receiver operating characteristic curve (ROC), and area under the receiver operating characteristic curve (AUC) [12]. However, overall model performance is generally evaluated by AUC in medical studies since both true/false positive and true/false negative are considered. Cross-validation is a resampling procedure used to evaluate ML models more objectively. We performed a five-fold cross-validation and the best model was chosen based on the highest AUC value. This was then used to develop a prediction system.

The Python programs in scikit.learn, Tensorflow, and other toolkits were utilized to complete the model building and relevant analysis [13]. The tools employed included Python 3.7.6, TensorFlow 2.1.0, Keras 2.3.1, Numpy 1.18.1, Pandas 1.0.3, imbalanced-learn 0.6.2, lightgbm 2.3.1, xgboost 1.1.1, matplotlib 3.1.3, and scikit-learn 0.22.2.post1.

### 3.5. Implementing an Interactive AI Prediction System with the Optimal Model

To verify whether the study’s optimal model was indeed feasible, the model was used to develop a prototype system with interactive functions for respiratory care members (physicians and respiratory therapists) to test and evaluate [9,14]. Based on Liao et al.’s study [15], we implemented this AI predictive system with web services technology in a digital dashboard form. The AI model was built with Python language and packed as a web service while the prediction system was developed with Microsoft Visual Studio^®^ and integrated into the HIS. The AI infrastructure in Chi Mei Medical Center includes one GPU (graphics processing unit) server for efficient training of models, one database server for storing big medical data, and one web-service server for storing the developed web services which can be used by HIS to perform real-time specific predictions.

According to the need for clinical respiratory therapy, this study set 11 time periods to provide timely predictions (each day was considered as a time period). A web-based dashboard system was designed to present the probability of successful weaning in each time period from the beginning of RCC admission to the nearest future time period for each patient. For example, if a patient stayed in RCC for 50 h, the dashboard would show the probability of the 24th, 48th, and 72nd hours. By observing the trend curve of the probabilities (colored balls), the respiratory care team had the capacity to objectively evaluate each patient and assess whether it was possible to try to wean them from ventilator use.

An important feature of our AI system is its ability to provide interactive functions, allowing respiratory care members to not only obtain current success probabilities quickly but also to manually adjust model feature values (e.g., SBP) to simulate the probability of success after possible changes in their patient’s overall health condition. This would be very helpful for advanced decision making and physician–patient communication.

## 4. Results

### 4.1. Demographics and Baseline Statistical Tests

After removing the missing or ambiguous values, a total of 670 patients who were admitted to the RCC at Chi Mei Medical Center between August 2016 and December 2019 were retrospectively included in the present study for building the AI model. The detailed baseline characteristics and grouping based on the success and failure of the weaning are shown in Table 1.

The results of the Spearman correlation analysis in Table 2 indicate the contribution of each feature to the outcome. It can be seen, based on the absolute values of the correlation coefficients, that the PSL had the strongest correlation with successful weaning outcomes, followed by FiO2, T-piece trial, mPaw, PEEP, and APACHE II score.

### 4.2. Modeling Results

**Figure 2:**
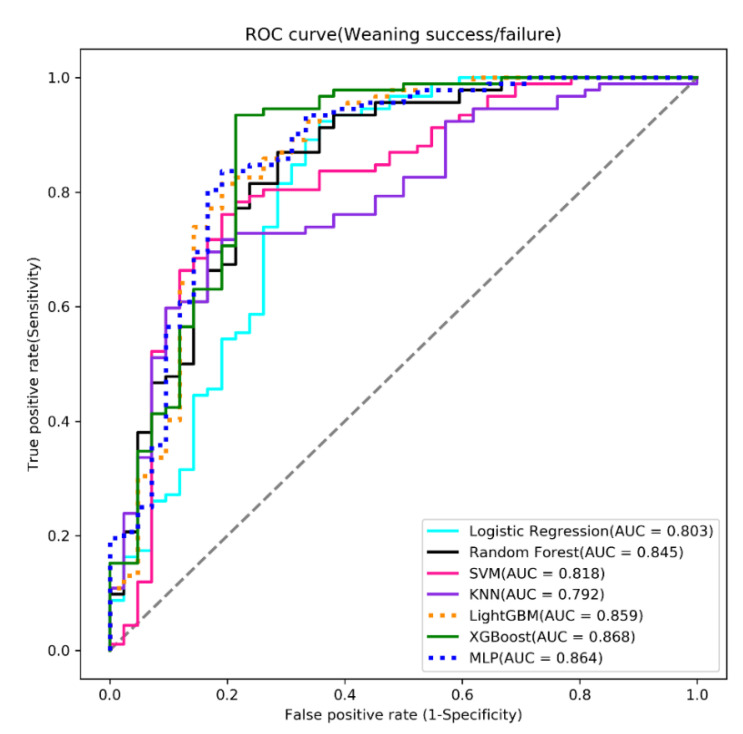
The receiver operating characteristic curve (ROC) of the testing results.

This study used seven machine-learning algorithms, namely LR, RF, SVM, KNN, lightGBM, XGBoost, and MLP, to build the prediction models with the training dataset. A grid search with fivefold cross-validation for hyper-parameter (see Table 3) tuning for each algorithm was conducted to obtain the best model. The prediction models were tested with the testing dataset and measured in terms of their accuracy, sensitivity, specificity, and AUC (see Table 4). Among the seven algorithms, the XGBoost algorithm had the best performance, with the highest AUC (see Table 4, Figure 2). Boosting algorithms are an advanced ensemble machine-learning strategy that endeavor to make an accurate classifier from various weak classifiers [16].

### 4.3. Prediction System Development and User Evaluation

**Figure 3:**
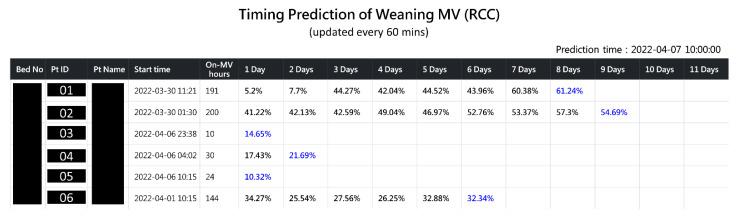
A screenshot of the AI prediction system (digital dashboard).

The XGBoost prediction model showed the best testing results among all the prediction models tested in this study and was therefore selected for subsequent clinical system development and deployment. The AI Center and Department of Information Systems of Chi Mei embedded the XGBoost model in a web-based system (digital dashboard) for predicting the optimal MV weaning timing for patients in the RCC (see Figure 3). The digital dashboard was launched in the RCC of Chi Mei Hospital in November 2020.

The digital dashboard was demonstrated to the RCC respiratory care members (three physicians and five therapists) and gained high recognition. They thought that it was a very useful tool in helping to decide whether it would be beneficial to try to wean patients from ventilator use. This decision could then be easily communicated with the patients or their families.

### 4.4. Use Case Scenario

As shown in Figure 3, the RCC respiratory care members applied the prediction system on the morning of 7 April 2022, to obtain an overview of the status of all the RCC patients. Patient 01 was admitted on 30 March 2022, having received 191 h of ventilator use after admission. For conservative consideration, the threshold for trying to wean was set at more than 50% in the initial stage of the deployment of the system (the threshold probability was initially set at 60%). This resulted in a probability of nearest future for successful weaning of 60.38% (day 7) and of nearest past of 61.24% (day 8). This showed that the patient’s respiratory ability had improved for two consecutive days (increasing probability of success) and demonstrated a higher value of successful weaning than the threshold set at 60%. Based on these data, the RCC respiratory care members (physicians and respiratory therapists) may have been able to successfully remove the patient from the ventilator on or before day 8. As for patients 03 to 06, the results showed that they were not suitable for weaning because of the low probability of success (<50%).

**Figure 4:**
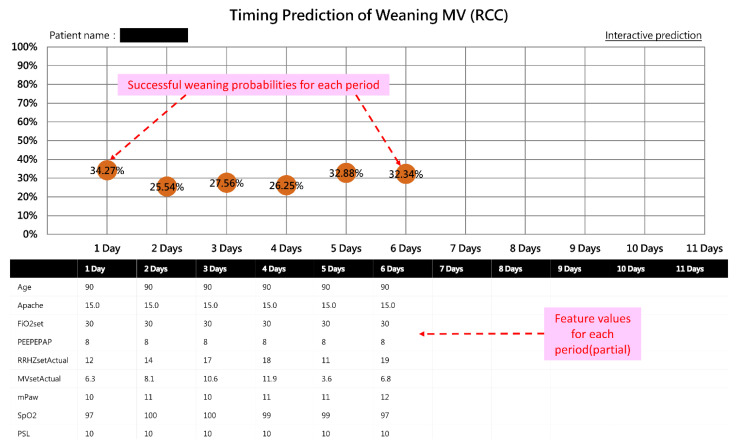
A screenshot of a specific patient’s curve chart of successful weaning probability.

**Figure 5:**
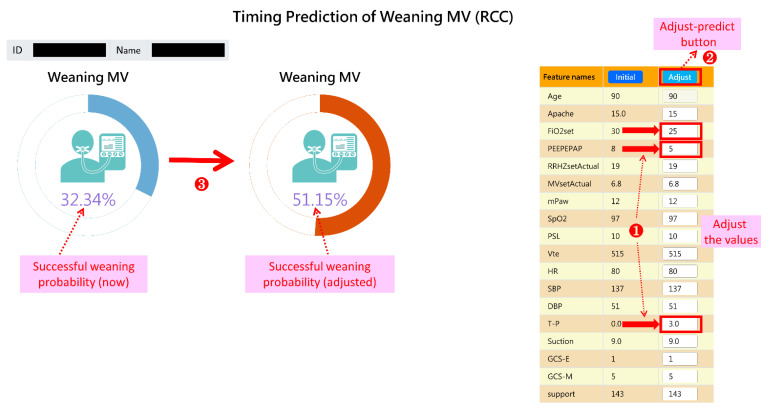
A screenshot of a specific patient’s interactive prediction.

Users can double-click a specific patient on the dashboard to see the curve chart of the probability of successful weaning and obtain an overview of the progress of the ventilator use (see Figure 4). As shown in Figure 4, users can click “Interactive prediction” to manually adjust the model feature values (e.g., FiO2set) and simulate the probability of success after possible changes in the patient’s overall health condition (followed steps 1, 2, 3 in Figure 5).

In this case, after the respiratory care members simulated the patient’s feature values of FiO2set (FiO2) from 30 to 25, PEEPEPAP (PEEP) from 8 to 5, and T-P trial (T-piece trial) from 0 to 3 (times), it was found that the probability rose from 32.34% to 51.15%, indicating that successful weaning could be performed based on the given health status. Therefore, the respiratory care members could strengthen the patient’s rehabilitation treatment to improve the patient’s respiratory muscle strength and endurance. Next, they could perform T-P trials and lower the FiO2 as soon as possible, so that the patient would be highly likely to successfully wean from the ventilator. Please note that part of the interface of the online dashboard is in Chinese, but these figures (Figure 3, Figure 4 and Figure 5) have been manually modified to English for an international readership.

### 4.5. Preliminary Impact Study for Patients

After the dashboard was launched in the RCC in late 2020, we obtained the mean intubation time of patients who were successfully weaned from MV during the initial adoption from January 2021 to March 2021. We then compared the results with those of the patients from the previous year (from January 2020 to March 2020 without AI assistance). Adult patients who had endotracheal intubation and had been removed from MV for 120 or more hours (i.e., successful weaning) were included in the comparison study. The analysis results (see Table 5) showed that the disease severity of the patients during AI intervention seemed greater than that of those without AI intervention. Further, the mean number of intubation days of those who were successfully weaned was reduced by 0.5 and the 120-hour successful weaning rate increased by 3.1%, indicating that our AI model did help patients wean from ventilators earlier, which could lead to a positive impact on quality of care.

## 5. Discussion

In this study, we built an AI predictive model and implemented an AI dashboard with the best model to help healthcare workers decide the best time to wean patients in the RCC. In addition to superior model performance, the preliminary impact analysis showed that the AI intervention reduced the number of intubation days for patients who were successfully weaned from MV and increased the weaning rate. To the best of our knowledge, no previous study has explored the development and use of AI to predict the successful weaning of patients from MV in the RCC setting. Thus, our study has profound academic and practical novelty and value.

The timing of extubation is important in mechanical ventilation. Delayed extubation prolongs hospital stays, increases medical costs, and results in greater suffering. Meanwhile, premature extubation increases the risk of extubation failure and re-intubation, which in turn leads to a higher risk of morbidity, greater medical expenditure, longer hospital length of stay, and increased risk of mortality [17]. Further, poor outcomes are associated with extubation failure. The early identification of patients’ ability to undergo a breathing trial can shorten their ventilator use duration. If a patient does not qualify for successful weaning based on the developed prediction system, premature extubation and extubation failure can be reduced, and alternative care practices can be instituted, such as an increased rehabilitation program, an evaluation of the patient’s nutritional status, and transfer to a long-term care facility to further reduce morbidity, length of hospital stay, and risk of mortality.

A recent study also used the electronic medical records of RCC patients in Taiwan from between 2013 and 2018 to develop a ML model to predict the successful weaning of patients from ventilators [18]. They used three models, XGBoost, RF, and LR, to establish the prediction model. The AUCS obtained were 0.908, 0.888, and 0.762 for the XGBoost model, RF model, and LR model, respectively. In the current study, seven supervised ML algorithms were used to build the model, and the AUCS obtained were 0.868, 0.845, and 0.803 for the XGBoost model, RF model, and LR model, respectively. In Lin’s study [18], the dataset and out-dataset contained similar clinical domains, which were: ventilation domain, physiology domain, laboratory domain, APACHE II score, and the presence of comorbidities. In the current study’s domain, the vital sign before weaning was used instead of the weekly average blood pressure, heart rate, body temperature, and oxygen saturation utilized in Lin’s study [18]. We also included the frequency of sputum suction in our analysis because airway hygiene plays an important role in successful weaning.

Lin’s study [18] used a total of 300 features (covering the average values of the same features of MV use during 1 to 6 weeks) to build an excellent prediction model. However, it may be difficult to actually apply it in clinics because it incorporates too many variables. In addition, the model may only be suitable for patients who have been on MV for 6 weeks (or more). However, based on general evidence, many RCC patients can be successfully weaned from MV before 6 weeks. By contrast, the present research aimed at practical usability and chose 26 features that are easily available in clinical practice to build a predictive model, and the predictable timing was not limited to the time of MV use. More importantly, we implemented the model as a predictive system (a digital dashboard) and demonstrated its feasibility and effectiveness.

A patient’s underlying disease could lead to respiratory failure; therefore, the disease should be treated and kept under control before weaning the patient from ventilator use in the ICU. The weaning procedure includes a gradual reduction in ventilator support by adjusting the ventilator setting and the oxygenation, making sure that the vital signs are stable during the process. The patient may then perform a spontaneous breathing trial and extubation. A study by Rose et al. [19] used knowledge-based automated weaning systems for the early detection of patients’ ability to spontaneously breathe and to inform decisions over the discontinuation of ventilation. These methods were found to reduce the duration of MV, the length of stay in the ICU, and the likelihood of tracheostomy for critically ill patients; however, a systematic review and meta-analysis showed no strong evidence of the effect on hospital stay, reintubation, self-extubation, non-invasive ventilation following extubation, or risk of mortality.

Ventilation is necessary to save lives, especially during respiratory failure, but prolonged use of ventilators increases the risk of several complications [20,21]. The weaning process accounts for about 40% of the total duration of ventilation [22]. With the improvement in information technology and the development of highly skilled organizations, both AI and ML have been widely applied in most healthcare settings. AI and ML methods have been found to exert a positive influence on patient outcomes. AI helps physicians to enhance their clinical decision processes by allowing them to efficiently use substantial amounts of digital information in ICU settings. A systematic review evaluated the efficacy and effectiveness of AI in weaning mechanically ventilated ICU patients [23]. AI application in the weaning process can be divided into ventilator and oxygenation management [23,24,25], spontaneous breathing trials [26,27,28], and extubation and arterial blood gas prediction [29,30]. It is worth mentioning that these applications rely on consulting systems that tend to operate according to less robustly confirmed methods. Further, it was found that it is difficult to test the system in different clinical situations. Because of the long list of decisions the algorithm makes, it is difficult to obtain ground-truth annotations from human experts. One criticism of AI is that its methods could lead to black-box results; that is, the algorithm can provide a physician with a prediction but cannot offer further information on how the prediction was made based on the given data. Efforts to explain AI are being made to enhance the clarity of its algorithms.

Before the era of AI, decisions over the weaning process and the timing of weaning were made based on the physician’s personal experience. Physicians take into consideration changes in vital signs, laboratory data, images, and ventilator settings to decide on weaning and extubation. Even after a comprehensive evaluation, weaning may be performed too late for some patients, and the risk of misjudgment still exists. The development of an AI model could support physicians’ decision making, allowing more precise and scientifically sound decisions over the weaning process.

There are some limitations to our study. First, the data used in the present study were obtained in only one medical center; thus, the results may not be generalized to other hospitals and areas. We call for further study by enrolling multiple centers. Next, we did not analyze medical images, such as computed tomography (CT) scans and chest roentgenography. We believe that the additional information from these images could further improve the prediction of the developed model. Finally, weaning is a dynamic process involving many confounding factors, which can change with time; therefore, some individualized factors need to be further evaluated.

## 6. Conclusions

Weaning strategies in RCC patients must be thoroughly planned, and determining the timing of weaning from ventilator use is one of the most important steps to consider. Incorporating a variety of clinically accessible factors that affect ventilator weaning in RCC, including patient physiology, nursing records, and mechanical ventilation parameters, this study used AI technology to develop an innovative system for predicting the optimal timing of weaning, which was integrated later on into clinical practice. A preliminary impact analysis showed its benefits, as expected. Based on these results, our study demonstrated the clinical usefulness of AI in RCCs. It is advised that future researchers consider including more parameters to improve the prediction accuracy of our study. A large-scale prospective study of AI intervention in MV weaning decision making in RCCs is needed. Moreover, the application of information technology infrastructure while implementing AI services in clinical settings is another critical issue. Advanced technologies, such as the Internet of Medical Things (IoMT), blockchain, and cloud computing should be studied [31,32].

## Figures and Tables

**Table 1:** Demographics and baseline statistical tests.

Feature	Total Patients	Weaning Failure	Weaning Success	*p*-Value
	N = 670	N = 210	N = 460	
Age, mean (SD)	68.9 (14.1)	70.0 (13.5)	68.4 (14.4)	0.161
Male, n (%)	409 (61.0)	140 (66.7)	269 (58.5)	0.054
APACHE II score, mean (SD)	16.1 (5.8)	17.9 (6.0)	15.3 (5.5)	<0.001
GCS_E, mean (SD)	3.2 (1.0)	3.0 (1.1)	3.3 (0.9)	0.005
GCS_M, mean (SD)	5.0 (1.0)	4.8 (1.2)	5.1 (0.9)	0.001
Diabetes, n (%)	261 (39.0)	93 (44.3)	168 (36.5)	0.068
COPD, n (%)	202 (30.1)	80 (38.1)	122 (26.5)	0.003
MI, n (%)	143 (21.3)	55 (26.2)	88 (19.1)	0.049
Stroke, n (%)	306 (45.7)	84 (40.0)	222 (48.3)	0.056
ESRD, n (%)	86 (12.8)	31 (14.8)	55 (12.0)	0.377
Pneumonia, n (%)	515 (76.9)	180 (85.7)	335 (72.8)	<0.001
Sepsis, n (%)	292 (43.6)	119 (56.7)	173 (37.6)	<0.001
HR, mean (SD)	87.4 (17.9)	92.8 (20.9)	85.0 (15.7)	<0.001
SBP, mean (SD)	127.9 (21.2)	126.9 (21.5)	128.3 (21.1)	0.452
DBP, mean (SD)	75.2 (14.8)	74.8 (14.5)	75.4 (14.9)	0.622
Frequency of suction (per day), mean (SD)	4.5 (5.7)	4.0 (5.1)	4.7 (5.9)	0.100
The duration of on-MV, mean (SD)	355.2 (209.2)	412.4 (267.2)	329.0 (170.6)	<0.001
FiO2, mean (SD)	25.8 (2.7)	26.8 (3.2)	25.4 (2.3)	<0.001
PEEP, mean (SD)	5.2 (0.8)	5.5 (1.0)	5.1 (0.5)	<0.001
RR Actual, mean (SD)	18.2 (5.5)	19.6 (6.0)	17.5 (5.1)	<0.001
MV Actual, mean (SD)	7.4 (2.6)	8.1 (2.9)	7.0 (2.3)	<0.001
mPaw, mean (SD)	8.6 (3.7)	9.5 (6.2)	8.1 (1.2)	0.002
SpO2, mean (SD)	98.4 (4.2)	98.2 (2.0)	98.4 (4.8)	0.451
PSL, mean (SD)	9.5 (2.1)	10.9 (2.7)	8.9 (1.3)	<0.001
PSL volume, mean (SD)	417.7 (115.7)	429.2 (126.8)	412.5 (110.1)	0.101
T-piece trial, mean (SD)	3.1 (4.1)	1.9 (3.6)	3.6 (4.2)	<0.001

Note1. APACHE II: Acute Physiology and Chronic Health Evaluation II; GCS_M, E: Glasgow Coma Scale—motor response, eye opening; COPD: chronic obstructive pulmonary disease; MI: myocardial infarction; ESRD: end-stage renal disease; HR: heart rate; SBP: systolic blood pressure; DBP: diastolic blood pressure; MV: mechanical ventilation; PEEP: positive end-expiratory pressure; RR Actual: respiratory rate, actual; MV Actual: minute ventilation, actual; PSL: pressure support level. Note2. *p*-value was examined by chi-squared test (categorical features) or two-sample t test (numerical features); null hypotheses: there are no differences among the demographic groups (variables).

**Table 2:** The correlation coefficient between each feature and outcome of successful weaning.

Feature	Correlation Coefficient	Feature	Correlation Coefficient
Age	−0.047	MV Actual	−0.186
Sex	−0.078	mPaw	−0.244
APACHE II score(RCC admission)	−0.208	SpO2	0.074
GCS_E	0.105	PSL	−0.410
GCS_M	0.109	PSL volume	−0.059
HR	−0.184	T−piece trial	0.245
SBP	0.042	Diabetes	−0.074
DBP	0.025	COPD	−0.117
frequency of suction (per day)	0.042	MI	−0.080
The duration of weaning	−0.140	Stroke	0.077
FiO2	−0.254	ESRD	−0.039
PEEP	−0.216	Pneumonia	−0.142
RR Actual	−0.160	Sepsis	−0.178

**Table 3:** Hyper-parameter range for experiments.

Method and Hyper-Parameter	Values
KNN	
weights	uniform, distance
n_neighbors (Number of neighbors)	range(1, 25)
algorithm	auto, ball_tree, kd_tree, brute
leaf_size	range(1, 5)
Logistic Regression	
penalty	l1, l2
C (Inverse of regularization strength)	1e−3, 1e−2, 1e−1
max_iter (Maximum number of iterations)	10, 30, 50, 100, 1000
SVM	
kernel	rbf, linear
gamma (Kernel coefficient)	auto, scale, 1e−2, 1e−3
C (Inverse of regularization strength)	1, 2, 5, 10
shrinking	True, False
Random Forest	
n_estimators (Number of trees in the forest)	100, 200, 500, 700, 1000
max_features	auto, sqrt
max_depth	auto, 15, 30, 50
LightGBM	
learning_rate	1e−3, 1e−2, 1e−1
num_iterations	100, 200, 500, 700, 1000
max_depth	4, 12, 15, 30, 50
num_leaves	1, 5, 10
feature_fraction	1e−1, 0.2, 0.5, 0.7
MLP	
hidden_layer_sizes	(100), (100, 55), (90, 60), (200, 150, 50),(64, 64, 32), (64, 128, 64, 32)
batch_size (Size of minibatches for stochastic optimizers)	8, 16, 32
learning_rate_init	1e−3, 1e−2, 1e−1
early_stopping	True, False
XGBoost	
learning_rate	1e−4, 1e−3, 1e−2
gamma (minimum loss reduction required to make a further partition on a leaf node of the tree)	1e−2, 1e−3, 1e−4, 1e−5
num_iterations	100, 200, 500, 700, 1000
max_depth	4, 15, 30, 50

Note. The hyper-parameters that are not described in this table were set to the default values used in the scikit-learn library.

**Table 4:** Testing results of the predictive models.

Algorithm	Accuracy	Sensitivity	Specificity	AUC
KNN	0.746	0.728	0.786	0.792
Logistic Regression	0.776	0.804	0.714	0.803
SVM	0.784	0.783	0.786	0.818
Random Forest	0.791	0.804	0.762	0.845
LightGBM	0.813	0.815	0.810	0.859
MLP	0.806	0.804	0.810	0.864
XGBoost	0.851	0.880	0.786	0.868

**Table 5:** The Preliminary Results of Clinical Evaluation and Comparison.

Indicators	(before AI Adoption) 2020/01–03 N = 57	(after AI Adoption) 2021/01–03 N = 58
RCC APACHE II score, mean	14.6	16.1
Intubation days of successful weaning, mean	16.7	16.2
120-h successful weaning-rate	67.1%	70.2%

## Data Availability

The dataset used for this study is available upon request from the corresponding author.
